# Switching it up: algal insights into sexual transitions

**DOI:** 10.1007/s00497-021-00417-0

**Published:** 2021-06-28

**Authors:** Susana M. Coelho, James Umen

**Affiliations:** 1 Max Planck Institute for Developmental Biology, Max-Planck-Ring 5, 72076 Tübingen, Germany; 2 Donald Danforth Plant Science Center, St. Louis, MO 63132 USA

**Keywords:** Sex determination, Sexes, Mating type, Reproduction, Mating system, Algae

## Abstract

While the process of meiosis is highly conserved across eukaryotes, the sexual systems that govern life cycle phase transitions are surprisingly labile. Switches between sexual systems have profound evolutionary and ecological consequences, in particular for plants, but our understanding of the fundamental mechanisms and ultimate causes underlying these transitions is still surprisingly incomplete. We explore here the idea that brown and green algae may be interesting comparative models that can increase our understanding of relevant processes in plant reproductive biology, from evolution of gamete dimorphism, gametogenesis, sex determination and transitions in sex-determining systems.

## Introduction

Sex is pervasive across eukaryotes and ensures the production of new genetic combinations. Although meiotic sex was established at the root of eukaryotes (Goodenough and Heitman 2014), a remarkable diversity of sexual characteristics has evolved in different lineages since then, including the way sexes or mating types are determined. Many species have the traditional male versus female dichotomy, but hermaphrodites or some combination of hermaphroditic and male–female differentiation occurs in others; some species determine sex genetically, often with highly differentiated chromosomes, while in others sex is determined by the environment or through an epigenetic mechanism. Intriguingly, modes of sex or mating-type determination and their integration into life cycles are evolutionarily labile within many lineages where transitions occur between genetic and epigenetic sex determination and in the phase of the life cycle (e.g., haploid versus diploid) in which sexual differentiation occurs (Box 1). Transitions among sexual systems have profound evolutionary and ecological consequences, influencing genetic diversity within populations, phenotypic evolution and patterns of diversification (Barrett 2010). For example, the switch from combined to separate sexes has occurred repeatedly in land plants and represents one of the major evolutionary transitions in their reproductive history. Understanding the proximate mechanisms, the ultimate causes and the consequences of transitions between sexual systems is major goals in evolutionary biology. However, we still know remarkably little about the diversity and evolution of sexual systems among plant taxa and about the mechanisms underlying transitions between reproductive systems. What are the molecular bases and the genomic consequences of shifts in sexual systems? Why do transitions occur more often in some groups than in others, and why do some sexual systems occur more frequently than others? What are the driving forces underlying transitions? These questions form a fascinating playground for current and future research. 

**Fig. 1:**
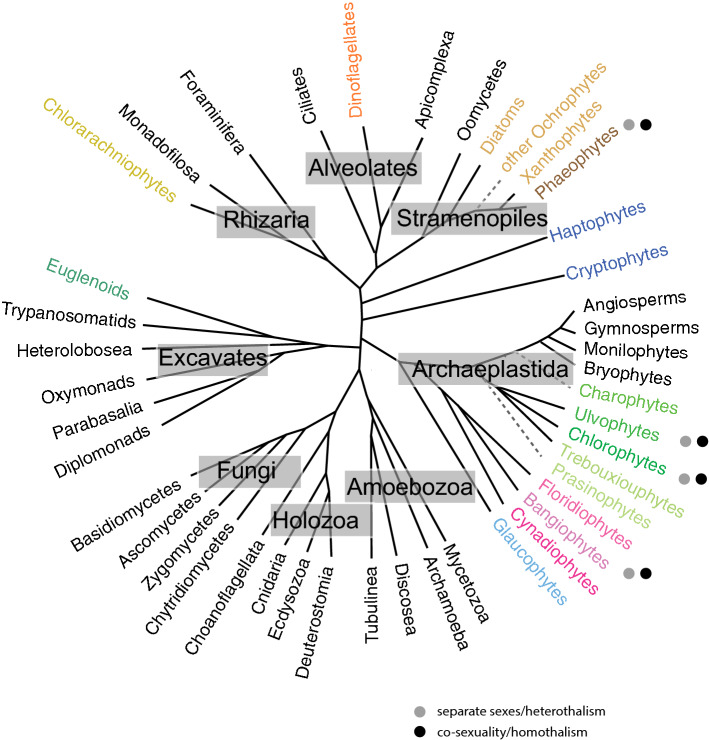
Cladogram of eukaryotes based on Coelho et al. (2018), highlighting groups containing algae (indicated by colored text at branch tips). Polyphyletic taxa, which are grouped for simplicity, are indicated by gray dashed lines

The increasing availability of large-scale sequencing and new technologies for investigating gene function have enabled new opportunities to develop non-classical plant models. Gametophyte-dominant land plants such as the bryophytes *Marchantia polymorpha*, *Ceratodon purpureus*, *Physcomitrella patens* (Chang et al. 2016) and green algae such as volvocines, *Closterium, Chara braunii*, *Ulva* sp. (Nishiyama et al. 2018; De Clerck et al. 2018; Tsuchikane and Sekimoto 2019; Umen and Coelho 2019; Bringloe et al. 2020) offer alternative systems to help answer questions on the mechanisms and evolution of sexual reproductive modes. In particular, some algae show comparable diversity and frequency of shifts in reproductive modes as land plants (Hanschen et al. 2018; Heesch et al. 2019; Tsuchikane and Sekimoto 2019). The diversity of reproductive systems in algae frame is important topic in a complementary way, and their elucidation will help generate a more balanced perspective on the origins and evolution of plant reproductive modes and provide more data for testing evolutionary theories about sex. It is important to note that while algae is not a taxonomically meaningful term, members of different algal groups circumscribe a large amount of eukaryotic diversity and, outside of fungi and metazoans, contain some of the best-studied sexual cycles among eukaryotes (Fig. 1). The green algae, specifically, are members of the Chloroplastida (green members of the Archaeplastida) and include Chlorophytes (e.g., volvocines, *Ulva*) as well as Streptophyte algae (e.g., *Chara, Closterium*).

Several recent reviews have addressed the details of sex and life cycles for different algal groups (Coelho et al. 2018; Umen and Coelho 2019; Coelho and Cock 2020) so we specifically focus here on how volvocine and brown algal models may be used to tackle questions of importance to plant reproductive biology, from evolution of gamete dimorphism, gametogenesis, sex determination and transitions in sex-determining systems.

## Volvocine algae

Volvocine green algae are members of the Chlorophyte algal lineage, a sister group to the Streptophytes (charophyte algae + land plants) from which they diverged around 1 billion years ago (Umen and Coelho 2019; Herron 2009). Moreover, they may offer insights into questions that are difficult to tackle in land plants such as the origins of anisogamy and oogamy, and the presence of deep homology in genetic control mechanisms for gamete differentiation and life cycle transitions. As described below, volvocines are also emerging as models for understanding transitions in sexuality from genetically determined sexes governed by sex chromosomes (heterothallism) to epigenetic sex determination (homothallism) where mitotic clones from a single individual can produce male and/or female gametes.

The volvocines have unicellular members, such as the well-studied model alga *Chlamydomonas reinhardtii*, and dozens of larger multicellular or colonial forms that are organized into genera based on cell number, morphology and organismal size. The most complex volvocine species are in the genus *Volvox* where individuals can have thousands of cells and exhibit full or partial germ-soma differentiation (Umen 2020). Molecular phylogenetic analyses have revealed that several volvocine genera, including *Volvox*, are polyphyletic (Herron and Michod 2008). While polyphyly complicates nomenclature, it makes the volvocine algae excellent models for comparative evolutionary studies of multicellularity and sexual cycles (Hanschen et al. 2018; Umen and Coelho 2019; Umen 2020).

Like most green algae and gametophyte-dominant land plants (e.g., bryophytes, lycophytes), volvocine green algae have haplontic life cycles where sex or mating type is determined in the haploid stage. In volvocine algae, the diploid stage is limited to a thick-walled and environmentally resistant zygotic spore. Key regulators of the haploid-to-diploid transition in Chlamydomonas and probably all volvocines are a pair of TALE-family homeobox transcription factors (TFs): KNOX-related Gsm1 and BELL-related Gsp1 which are expressed in *minus* (*GSM1*) or *plus* (*GSP1*) gametes, respectively. Upon fertilization, Gsm1 and Gsp1 heterodimerize, enter the nucleus and activate the zygotic (sporophytic) differentiation program (Lee et al. 2008; Hamaji et al. 2016; Kariyawasam et al. 2019a). This mechanism may help ensure that the diploid or sporophytic differentiation program is only activated upon fertilization (Haag 2007; Perrin 2012) and is conserved across a range of eukaryotes including bryophytes, brown algae, fungi and cellular slime molds (Bloomfield 2019).

Genetic mechanisms for mating-type or sex determination in volvocine algae have been investigated extensively and have yielded insights that may be applicable to understanding sex determination in other green algal taxa and land plants (Umen and Coelho 2019). In *C. reinhardtii* and other heterothallic (dioicous) volvocine algae, the master regulatory gene *MID* gene is present in the *minus* mating type locus (MTL) or male sex-determining region (SDR). Expression of *MID* is sufficient to cause *minus* or male gametic differentiation, while its absence results in the default state of *plus* or female differentiation (Umen and Coelho 2019). The Mid protein is an RWP-RK family TF, and relatives from this family are now known or suspected to play a role in mating type or sex determination in more distantly related green algal taxa (Yamazaki et al. 2017; Blanc-Mathieu et al. 2017) as well as in land plants (Hisanaga et al. 2019). In the liverwort *Marchantia polymorpha*, an RWP protein encoded by the MpRKD gene is required for both male and female gametogenesis (Koi et al. 2016; Rövekamp et al. 2016) while in the algae there is a single gene in one of the two sexes or mating type loci, prompting the question of whether the roles of RWP-RK TFs are conserved or there has been some modification and/or divergence between the streptophyte and chlorophyte lineages in their genetic circuitry for gamete differentiation.

The evolutionary histories and relatedness of MTL and SDRs in volvocine algae have been reviewed recently (Umen and Coelho 2019). These regions of heterothallic volvocine species can span > 1 Mbp and share several properties in common with each other including suppressed recombination between the two MTL or SDR haplotypes, rearrangements that disrupt sequence collinearity, presence of gametologs (genes with an allele in both haplotypes) and sex-limited genes such as *MID* that are found in only one of the two haplotypes. In *Volvox carteri*, the SDR haplotypes are highly differentiated and expanded in size with respect to MTL/SDR regions of other volvocine genera and have increased repeat content that is characteristic of non-recombining heteromorphic sex chromosomes from other taxa including plants and some multicellular algae such as Ulva and *Ectocarpus* (Coelho et al. 2018). Unlike what has been found for other volvocine genera, the gametologs in *V. carteri* are also highly diverged due to absence of recombination, and at least some of these SDR genes are likely to have undergone sexually antagonistic selection (Ferris et al. 2010; Geng et al. 2014; De Hoff et al. 2013; Hamaji et al. 2018).

**Fig. 2:**
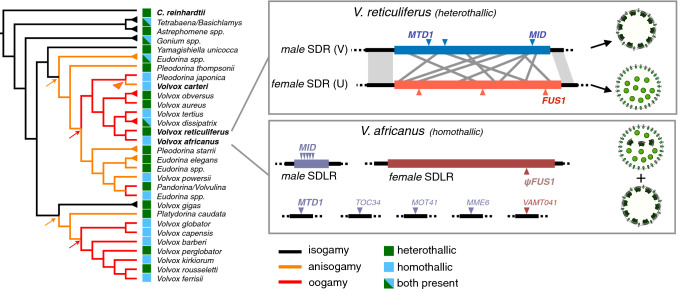
Left side, cladogram of volvocine algae with unicellular outgroup species *Chlamydomonas reinhardtii* at top. Remaining species or groups are multicellular. Branches are colored based on degree of sexual dimorphism, with black for isogamy, orange for anisogamy and red for oogamy, with possible transitions noted by arrows or arrowheads. Branches terminating in triangles represent multiple independent isolates. Sex determination system is shown by colored squares with green for heterothallic, blue for homothallic, and mixed when both are found. Box in upper right depicts the male (V chromosome) and female (U chromosome) sex-determining regions (SDRs) of *Volvox reticuliferus *U and V sex chromosomes. Blue and red are the non-recombining heteromorphic portions of each haplotype with rearranged gametologs represented by gray connecting lines, and sex-limited genes by triangles. Conserved genes *MID*, *MTD1* and *FUS1* are marked. Cartoons show sperm packet bearing male or egg-bearing female sexual phase spheroids whose development is governed by the male or female SDR. Lower box depicts chromosomal regions of homothallic species *Volvox africanus* with male and female SDR-like regions (SDLRs) colored dark blue and dark pink, respectively. SDR derived genes (former gametologs) that inserted into different autosomal regions are shown below, with male SDR derived genes in blue and female derived SDR genes in pink. Cartoons depict the two types of sexual individuals produced from *V. africanus* clones—male spheroids and monoicous spheroids containing eggs and sperm packets. This figure was based on previously published work (Umen and Coelho 2019; Yamamoto et al. 2021)

Transitions between heterothallism and homothallism (or vice versa) have occurred in most volvocine genera, often multiple times (Fig. 2) (Hanschen et al. 2017) but until recently had not been investigated in detail. Hints about how such transitions could arise have come from studies in heterothallic species *C. reinhardtii* and *V. carteri* where mutations or genetic manipulations can cause a self-mating phenotype. In *C. reinhardtii*, the autosomal *iso1* mutation caused gametes to self-agglutinate when *iso1* was in a *MT-* strain background, though the causative mutation has not been identified (Campbell et al. 1995). A *C*. *reinhardtii MT* + strain with an extra independently assorting and unstable copy of *MID* was also found to cause a self-agglutinating phenotype, presumably due to stochastic mitotic loss of the *MID* gene (Ferris and Goodenough 1997). In *V. carteri* males, a strong RNAi-mediated knockdown of *MID* caused differentiation of sperm precursor cells into partly functional and fertilizable eggs, while a partial knockdown of *MID* led to a co-sexual phenotype where a single sexually induced individual produced both sperm and eggs and displayed self-fertility (Geng et al. 2014). These studies highlight what appears to be an intrinsic bi-stability in volvocine algal gamete differentiation. This is clearest in the case of *V. carteri* where gamete precursor cells with a partial *MID* knockdown adopted either a male of female identity rather than showing some combination of the two sexes and/or infertility. Thus, by adding a stochastic component to *MID* expression or activity, the sex determination system in volvocine algae can be pushed toward its intrinsic bistable tipping point and result in a transition from a strictly male phenotype to homothallism. Indeed, a study of the homothallic *Volvox* species *V. africanus*, which produces either male-only individuals bearing sperm packets or co-sexual individuals containing both eggs and sperm packets (Fig. 2), revealed that a *MID* gene was present and that its expression level was correlated with the degree of male differentiation in each of the two forms (Yamamoto et al. 2017).

The transition between heterothallic and homothallic mating in Volvox poses additional questions related to how sex chromosome differentiation and sex determination systems interact and the fitness consequences of going from outcrossing to selfing (Hanschen et al. 2017). In *V. carteri*, Mid controls most of the gamete cell differentiation program, but there are some aspects of fertility and sexual differentiation that are under the control of the sex chromosomes but not the Mid pathway, and these features are revealed when SDR haplotype and Mid pathway expression are mis-matched (Geng et al. 2014). Genes that control these additional male and female reproductive fitness traits may be gametologs that have undergone sexually antagonistic selection, or male-/female-specific SDR genes. This finding begs the question of how male-specific or female-specific functions that evolved as sex chromosome genes become resolved in a transition to homothallism? A recent study comparing the genomes of homothallic *V. africanus* to male and female SDRs of a heterothallic close relative, *V. reticuliferus* (~ 11 My divergence time), has begun to shed light on this question (Yamamoto et al 2021). Although *V. africanus* no longer possesses sex chromosomes, it retained a chromosomal region that was very similar in size, gene content, and sequence characteristics to the *V. reticuliferus* female SDR, revealing a past history of this SD-like region (SDLR) as a female sex chromosome (Fig. 2). Notably, while many of the female gametolog descendants were retained in the SDLR of *V. africanus*, no homologs of female sex-limited genes were retained, including the conserved *FUS1* gene encoding a membrane-localized fertilization protein that was also lost in the *V. carteri* lineage. Importantly, as predicted from earlier work (Yamamoto et al. 2017), *V. africanus* had a separate chromosomal region that contained a tandem array of *MID* genes and an unlinked autosomal region containing another conserved male/minus gene, *MTD1*. Interestingly, three other autosomal regions had insertions of male-derived gametologs and one had a female gametolog. Moreover, there were no retained pairs of ancestral gametologs in *V. africanus*—most were derived from the ancestral female SDR and remained in the SDLR. Thus, it appears that the retention of male–female gametolog pairs was not tolerated or required for homothallic sex determination. However, the ancestral SDR haplotype for retained gametologs might still be important for sex determination or reproductive fitness. Intriguingly, one of the three retained male ancestral gametologs in *V. africanus*, *MOT41*, encodes intraflagellar transport protein IFT43 with a predicted motility-related function (Taschner and Lorentzen 2016) hinting at a possible case of sexual antagonism where the male allele was required in *V. africanus* due to its adaptation for male fitness in sperm. The converse may be true for some of the ancestrally female gametologs in the SDLR of *V. africanus*. While these hypotheses about selective gametolog retention remain to be tested, the genomic analysis of homothallism in *V. africanus* and sexuality in the genus *Volvox* have opened the door to answering these and others about how formerly male-adapted and female-adapted genes can be lost, gained and/or possibly reshaped by selection during a transition from genetically determined sexes to co-sexuality. At the same time, there are also opportunities for investigating the ecological significance of these transitions in volvocine algae and now they impact (or are impacted by) organismal size/complexity, population structure and the environment.

## The brown algae: distant comparative models

The brown algae (Phaeophyceae) belong to the stramenopile (or heterokont) supergroup and have had a very different evolutionary history than the green algae. The ancestor of stramenopiles diverged from the Archaeplastida lineage (Viridiplantae, Glaucophyta, Rhodophyta) and other eukaryotic lineages near the base of the eukaryotic crown radiation (Bringloe et al. 2020). Brown algae represent the third most complex multicellular lineage on the planet (Cock et al. 2010).

Brown algae have been used for decades to investigate early embryogenesis (reviewed in Brownlee et al. 2001; Coelho and Cock 2020) because fertilization is external, and therefore, zygotic development can be easily followed. In recent years, the development of filamentous brown algae from the genus *Ectocarpus* as a model has significantly contributed to increase our understanding of the molecular bases for reproductive evolution in this group of eukaryotes. Several sexual life cycle master regulators have been identified and characterized in *Ectocarpus* (Coelho et al. 2011; Arun et al. 2019), and the chromosomal basis of sex determination has been described (Ahmed et al. 2014; Luthringer et al. 2015b; Coelho et al. 2018) (Lipinska et al. 2017, 2019). *Ectocarpus* has a U/V sex determination system where sexes are determined at meiosis, and expressed during the haploid (gametophyte) stage of the life cycle. The presence of a V (male) sex chromosome in spores triggers the male gametophyte developmental program, whereas spores that inherit a U chromosome become female gametophytes. The U and V sex-specific regions of *Ectocarpus* stopped recombining at least 160 MY ago, and a group of genes was shown to be conservatively sex-linked across a range of brown algae (Lipinska et al. 2017), suggesting that brown algae share an ancestral U/V sex chromosome. The *Ectocarpus* V sex chromosome is dominant over the U (Ahmed et al. 2014), and it is thought that maleness is determined by a masculinising factor located on the V-specific region. Female sex is determined in the absence of this factor. However, recent work using the giant kelp *Macrocystis pyrifera* has shown that although female morphological features can be expressed in the absence of the U chromosome, the U-specific region may be required to fully express the female developmental program (Müller et al. 2021).

The brown algae are fascinating comparative models for investigating the evolution and regulation of sexual life cycles and reproductive characters with relevance to other eukaryotic lineages (Coelho et al. 2018, 2020; Coelho and Cock 2020). Like some green algae (e.g., *Ulva *spp.) and land plants such as ferns, many brown algae have haplo-diplontic life cycles where sex is determined in the haploid stage (Luthringer et al. 2015a). Consistent with the inferred old age of the *Ectocarpus*, suppression of recombination event between the U and the V, phylogenetic analysis and ancestral state reconstructions suggest that haploid genotypic sex determination is the ancestral state in this lineage (Heesch et al. 2019). These observations highlight the deep evolutionary roots of genetic sex determination in this group of organisms and pose interesting questions on what leads to different degrees of conservation versus turnover for sex chromosomes in different eukaryotic lineages (Beukeboom and Perrin 2015).

**Fig. 3:**
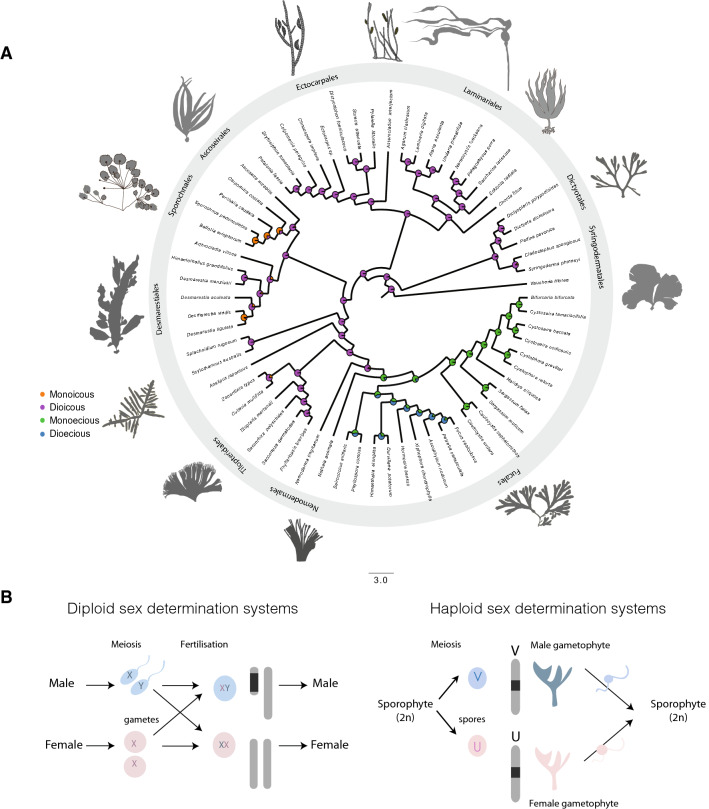
**a** Maximum likelihood ancestral state reconstruction based on Heesch et al. (2019) for brown algal sexual systems. Pie charts and colors at each node represent the probabilities for each state. Colors at the tips represent the species states. **b** Schematic view of diploid (XX/XY) and haploid (U/V) genetic sex determination systems

The use of correlative phylogenetic approaches to investigate the evolution of reproductive characters in the brown algae has revealed a complex evolutionary history of sexual and life cycle traits (Heesch et al. 2019). In this group, sex determination and sexual differentiation may occur during either the haploid or the diploid phase of the life cycle, by genetic or epigenetic mechanisms, and the level of sexual dimorphism varies considerably across species. The remarkable diversity of sexual traits, including multiple transitions between sexual systems over a relatively short evolutionary time period (less than 200 my) (Coelho et al. 2018; Heesch et al. 2019), are exceptional among eukaryotes and make the brown algae of interest to study the mechanisms underlying transitions between sexual systems without being obscured by large evolutionary times (Fig. 3).

**Table:** 

Box 1: Life cycle complexity and sexual reproduction	
Sexual reproduction is inherently linked to life cycle. Meiosis and gamete fusion (fertilization) alternate during sexual life cycles. Several types of life cycles exist in nature (Coelho et al. 2007, 2018). Animals have diplontic life cycles where sex is determined in the diploid phase and somatic development of a diploid zygote produces the adult organism. In diplontic life cycles, the only representatives of the haploid phase are the unicellular gametes. Many eukaryotic taxa such as many green algae have haplontic life cycles where cell divisions (somatic/vegetative development) and sex determination (or mating-type determination) occur during the haploid phase and the diploid phase is reduced to a resting spore which eventually will undergo meiosis to produce new haploid progeny. In haplo-diplontic life cycles, mitotic cell divisions occur during the haploid and the diploid stages to produce haploid or diploid adult forms that may be similar or different from each other in morphology. In haplo-diplontic systems, sex may be determined in either the haploid or diploid phase of the life cycle. Many plants and some algae have complex life cycles involving alternation between a gametophyte generation and a sporophyte generation. Usually, gametophytes are haploid and sporophytes are diploid, but this is not a strict rule, and many examples exist where ploidy and generation stage are uncoupled (Bothwell et al. 2010; Coelho et al. 2011). Recent work has shed some light on the genetic and epigenetic mechanisms regulating life cycle switches in organisms with complex life cycles (e.g., Sakakibara et al. 2013; Arun et al. 2019; Bourdareau et al. 2021; Borg et al. 2021). A consequence of variation in the types of life cycles is that genders may be determined both during the haploid stage (in haploid-diploid and haploid life cycles) or during the diploid stage (in organisms with diploid (or haploid-diploid life cycles) (Fig. 3). Moreover, sexes may be determined by genetic or epigenetic factors.	

The transition from haploid to diploid sex determination represented a major event during the evolution of many eukaryotic lineages, being associated, for example, with the origin of vascular plants and diploid-dominant life cycles (Villarreal and Renner 2013). However, the underlying mechanisms and ultimate forces that drove this important evolutionary transition are elusive. It is believed that diploid genetic sex determination systems (XY or ZW) did not evolve directly from haploid sex chromosome-based systems (U/V) but that this transition required intermediate stages with epigenetic (developmental) sex determination during which the timing of sexual differentiation shifted from the haploid to the diploid phase (Beukeboom and Perrin 2015). Although this idea is consistent with the phylogenetically based trends in the green and the brown lineages, there are currently no empirical studies addressing this key evolutionary question. Notably, in well-studied models such as the green alga *Chlamydomonas reinhardtii*, a vegetative diploid phase can be artificially intercalated into what is normally a dormant stage of the life cycle (Kariyawasam et al. 2019b), so this route may have been available in naturally occurring plant systems. Land plants also include species with both haploid and diploid sexual systems, but the events underlying transitions are difficult to study because of the large evolutionary distances (> 500 MY (Renner et al. 2017) between taxa with U/V systems [e.g., *Ceratodon *(McDaniel et al. 2013b)] and taxa with diploid (XY or ZW) sexual systems (e.g., Silene (Charlesworth 2014, 2016; Bergero et al. 2015; Krasovec et al. 2018). The brown algae are attractive in this respect because the U/V > XY transition occurred within the last 175 MY (Silberfeld et al. 2010). On-going projects aimed at sequencing brown algal species across the whole phylogeny and, in particular, brown algae that have XY systems and outgroups with U/V systems will be instrumental to tackle the molecular events that underlie this transition and will illuminate the evolutionary trajectory of the transition from U/V toward XY/ZW sexual systems.

While much effort has been invested into studying why and how dioecy/dioicy and sex chromosomes emerged repeatedly in land plants (McDaniel et al. 2013a; Charlesworth 2016), the questions of how and why dioecy evolves toward co-sexuality have been largely overlooked (Kafer et al. 2017). When brown algal sexual systems are mapped onto a phylogenetic tree, the distribution indicates that there has been considerable switching between sexual systems during evolution (Silberfeld et al. 2010; Heesch et al. 2019). Dioicy (separate sexes during the haploid phase) appears to have been the ancestral state, but there are many extant co-sexual species with haploid sexual systems (i.e., monoicous species). Moreover, following the transition to diploid sex determination in the order Fucales, this order also diversified to include species with separate sexes (dioecious) and co-sexual (monoecious) species (Silberfeld et al. 2010; Cánovas et al. 2011). The Fucales have undergone considerable turnover in terms of diploid sexual system states, with several independent switches between dioecy and monoecy having occurred in closely related species (Cánovas et al. 2011). The Fucales therefore represent interesting systems to elucidate the mechanistic and evolutionary bases of transitions between separate and combined sexes during the diploid phase of the life cycle and the dynamics of diploid sex chromosome evolution. Note that analysis of the mechanisms underlying switches from dioecy to co-sexuality is challenging in plants because genomic information, including knowledge of sex chromosome and sex determination mechanisms for pairs of dioecious–monoecious species, is relatively limited. Models predict that the probability of breakdown of dioecy and transition to co-sexuality occurring will depend on the sexual system of the dioecious ancestor, specifically the age and extent of degeneration of the sex chromosomes, with transitions toward co-sexuality being more likely when the non-recombining sex chromosomes are not highly degraded [e.g., (Ehlers and Bataillon 2007; Crossman and Charlesworth 2014)]. These predictions have the possibility to be tested by accessing to the features of several XY or ZW systems and focusing on pairs of species that reverted to monoecy.

## Conclusion and outlook

Volvocine algae have long been recognized as models for eukaryotic sexual cycles and the evolution of anisogamy. With growing numbers of sequenced volvocine genomes, including both homothallic and heterothallic species, it is now possible to begin reconstructing molecular events related to transitions in sexuality. Some promising avenues of investigation are how master regulatory gene *MID* expression is modulated in homothallic species, and how formerly masculinized and feminized portions of heterothallic genomes respond to an altered selective landscape when they must coexist in the same genome after a transition to homothallism.

Although the brown algae have been evolving independently from land plants for millions of years, they are interesting comparative models because both groups share common, convergent features in terms of their sexual systems, such as the presence of U/V sex chromosomes and the existence of many transitions between separate sexes and co-sexuality. Recent progress in the development of genetic and genomic tools provides a solid foundation for the future advances of brown algal developmental biology and comparative molecular biology. For example, on-going projects aimed at sequencing species across the whole brown algal phylogeny and, in particular, brown algae that have XY systems and outgroups with U/V systems will be instrumental to tackle the molecular events that underlie the transition U/V towards XY/ZW sexual systems.
